# Intrinsic Effect of Pulsed Current on the Recrystallization of Deformed AZ31 Alloy

**DOI:** 10.3390/ma15072698

**Published:** 2022-04-06

**Authors:** Jie Wu, Xiaobo Wang

**Affiliations:** 1 College of Architecture, Taiyuan University of Technology, Taiyuan 030024, China; wjielyf@163.com; 2 Department of Physics and Electronic Engineering, Jinzhong University, Jinzhong 030619, China

**Keywords:** recrystallization, alloys, pulsed current

## Abstract

Two ensemble configurations were designed to investigate the intrinsic effect of a pulsed current on the recrystallization of rolled AZ31 alloy. The samples with a total reduction of about 60% were crystallized at 473K for 5 min when treated with the pulsed current. By forcing the pulsed current flow only through the graphite die, and the sample was heated by Joule effect, a microstructure with a grain size of ~5 μm was formed and the recrystallized fraction achieved 60% reduction. Moreover, a fully recrystallized microstructure with a grain size of ~9 μm was obtained when heated with Joule and athermal effects by forcing the pulsed current flow through the sample only. Based on the experimental results, the recrystallization behavior of deformed AZ31 under a pulsed current should be governed by the high Joule heating effect, which could generate transient high stress in the sample due to the nonsynchronous change in temperature and thermal expansion. The athermal effect of the pulsed current could enhance the dislocation mobility and thus accelerate coarsening of the recrystallization grains, but it should not be the key factor governing the recrystallization behavior of rolled AZ31B. This led to the p erroneous conclusion that the athermal effect of pulsed current played a crucial role in the recrystallization of deformed alloys.

## 1. Introduction

Pulsed currents have been applied for improving the deformation limit and controlling the microstructure of difficult-to-deform materials by grain refinement and deformation resistance reduction [1,2,3]. To date, numerous materials, including metals and alloys, intermetallic compounds and ceramics with improvements in mechanical properties, microstructure, and other special properties have been successfully fabricated using pulsed currents [4,5,6,7]. At the present level of understanding, the enhancements in properties are attributed to athermal effects including the reduction in activation energy for defects [8], an increase in defect concentration [9], and electron wind modifications of the diffusion flux [10].

However, the influence of high heating rates provided by the pulsed current was neglected, which could generate high transient stress in the sample due to the nonsynchronous changes in temperature and thermal expansion [11]. As the high heating speeds were induced by the Joule heating effect of the pulsed current, the effect of Joule heating cannot unambiguously be separated from the intrinsic role of the pulsed current. Through experimental investigation and theoretical analysis, Liu [12] concluded that the athermal effects of pulsed current were considered a key role governing the recrystallization of the deformed alloys. Careful examination of the experimental procedure could find that the extremely high heating rates of pulsed currents have not been taken into account while analyzing the results. Thus, the general advantages of pulsed currents have not been proved to be an intrinsic (i.e., non-thermal) effect or a result of the high heating speeds achievable through the Joule heating effect.

To circumvent this issue, the ensemble configuration should be carefully designed to guarantee that all the parameters are absolutely the same in all experimental modes. In the present study, two ensemble configurations were designed to investigate the high heating speeds achievable through the Joule heating effect of pulsed currents on the recrystallization of deformed AZ31B alloys. These results will contribute to the fundamental understanding of the pulsed current and, in turn, guide the rational use of pulsed currents to produce materials with controlled microstructures and tunable properties.

## 2. Experimental Section

The as-received alloy was a commercial hot-rolled sheet of AZ31 Mg alloy provided by Maige Co., Ltd., Shanghai, China (3.1 wt. %Al, 0.9 wt. %Zn, 0.4 wt. %Mn, balance Mg) with a thickness of 15 mm. Prior to the warm-rolling, the sheet was homogenized at 350 °C for 1 h. The main proposed application of homogeneous annealing is to prevent cracking during subsequent warm-rolling. Thus, the sheet is rolled with processing parameters as follows: rolling temperature of 200 °C, thickness reduction of 60% and 5 passes. The final thickness of the rolled strip after rolling was about 6 mm. Samples with a diameter of 20 mm were cut from the rolled strip by electro-discharge machining (EDM, Keyi Co., Ltd., Shenyang, China) for subsequent use.

**Figure 1:**
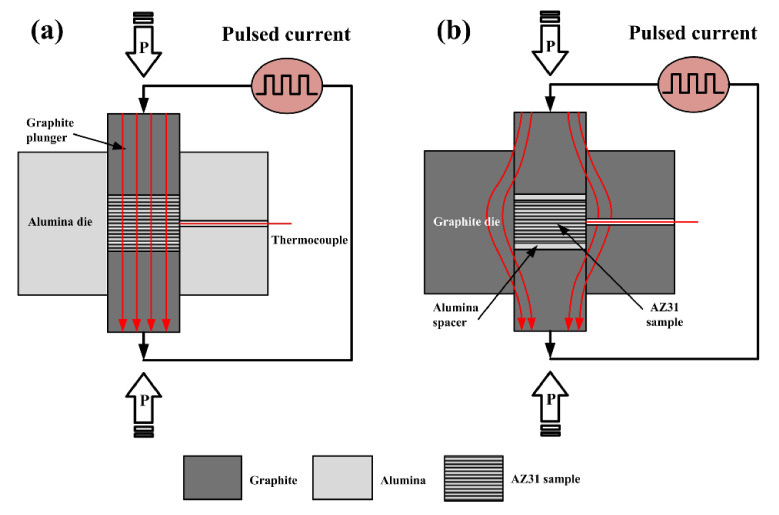
Schematic representation of the ensemble configurations: (**a**) current-assisted mode; (**b**) current-insulated mode.

Spark plasma sintering (which is often abbreviated to SPS) apparatus (Model 1050, Sumitomo Coal Mining Co., Ltd., Tokyo, Japan) with an on/off ratio of 12:2 and pulse duration of 3.3 ms was used to supply the pulsed current. The uniaxial pressure was set as low as 20 MPa for all experiments to minimize the effect of mechanical pressure on the recrystallization behavior, which could mask the intrinsic effect of the pulsed current. To separate the Joule and non-thermal effects of the pulsed current during SPS, two ensemble configurations were carefully designed, as shown in Figure 1. In the current-assisted mode shown in Figure 1a, the pulsed current is forced to pass only through the sample by using an alumina die. In this case, the sample is heated by both the Joule heat and athermal effects of the pulsed current. For the current-insulated mode shown in Figure 1b, the electrically isolative thin disks of alumina with a thickness of 1 mm were used to isolate the sample from the graphite punches; thus, no current was allowed to flow through the sample. In this case, the sample was heated by the thermal conductivity of graphite punches and alumina disks. The samples treated with the current-assisted and current-insulated modes were denoted as CA and CI in the present study, respectively.

The samples were taken from the cross-section along the compression direction to prepare samples for electron back scatter diffraction (EBSD). The texture of the rolled samples was measured using Cu Kα radiation in a Panalytical Xpert Pro MRD diffractometer equipped with a PW3050/60 goniometer (PANalytical, Almelo, The Netherlands). Scanning electron microscopy apparatus (SEM, JSM-6700F, JEOL Ltd., Tokyo, Japan) equipped with an Oxford detector was employed for the EBSD measurements. The step size was 0.2 μm on all the EBSD scans. HKL Channel 5 (HKL Technology, Hobro, DK-9500, Denmark, 2001) software was used for the EBSD analyses.

## 3. Results and Discussion

**Figure 2:**
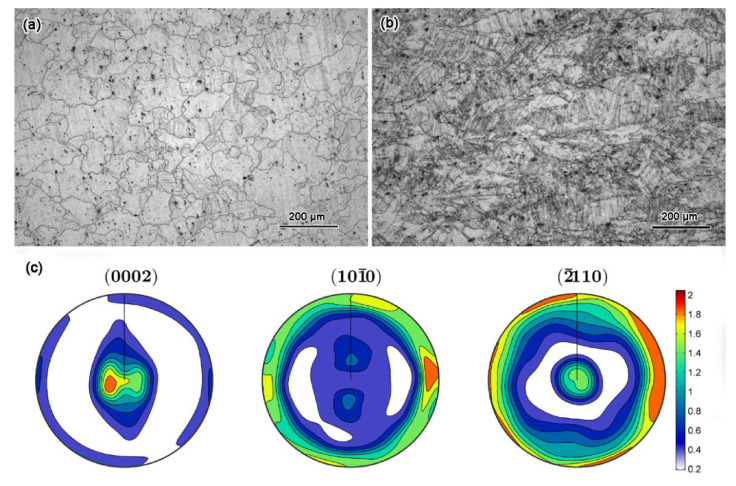
OM images of the homogenized (**a**) and as-rolled (**b**) AZ31 alloy, and the pole figures of the as-rolled sample (**c**).

Figure 2a,b show the OM images of the homogenized and as-rolled AZ31 alloy. After homogenizing heat treatment, the AZ31 was consisted of coarse equiaxed grains. No residual twins and deformed structures were detected. The average grain size was measured as 70 μm. However, the grains were elongated along the rolling direction after the warm-rolling. Meanwhile, many deformation twins and shear bands were observed in the grains, as shown in Figure 2b. In addition, the as-rolled sample shows a strong (0001) [11,12,13,14,15,16,17,18,19,20] texture as shown in Figure 2c.

**Figure 3:**
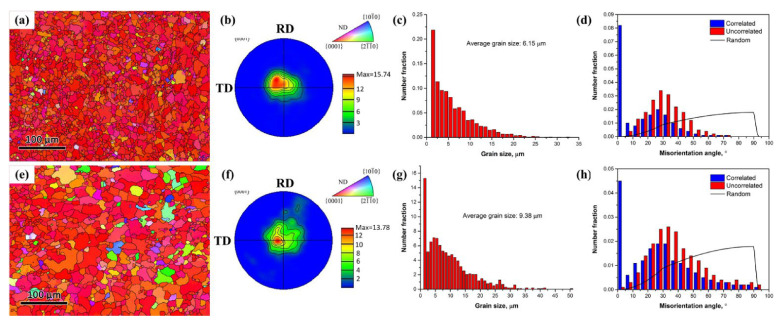
EBSD images, (0002) pole figure, IPF map, distribution histogram of grain size and misorientation distribution of the CI and CA samples: (**a**–**d**) the CI sample; (**e**–**h**) the CA sample.

Figure 3 shows EBSD images, (0002) pole figure and inverse pole figure (IPF) map, distribution histogram of grain size and misorientation distribution of the CI and CA samples on the rolling-transverse (RD-TD) plane. It has been reported that at least 30 min was needed to form a few recrystallized grains at 210 °C in the AZ31, even with a reduction of 61% [13]. By heating with the pulsed current, both the CI and CA samples exhibited homogeneous and fine grains without twins (Figure 3a,e). The average grain sizes of the CI and CA samples were 6.15 μm and 9.38 μm (Figure 3c,g), respectively. However, the texture intensity and misorientation distribution were barely changed with and without the pulsed current. The CI and CA samples exhibited strong basal texture intensities of 15.74 and 13.78 with in c-axis, with the majority of the grains being parallel to normal direction (ND) (Figure 3b,f). The misorientation angles of around 1^°^ from neighboring points account for the largest fraction.

**Figure 4:**
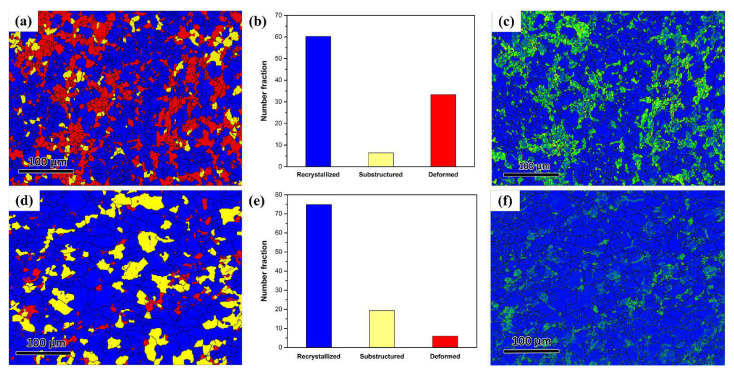
Recrystallized, substructured and deformed fraction as well as the KAM map of the CI and CA samples: (**a**–**c**) the CI sample; (**d**–**f**) the CA sample.

Figure 4 shows the recrystallized, substructured and deformed fraction as well as the kernel average misorientation (KAM) map of the CI and CA samples. The KAM value is a measure of local grain misorientation which is usually derived from EBSD data. The KAM value can be used to represent the homogeneity of plastic deformation. The areas with large KAM values suggest high plastic deformation or high-level stress states. The stress in recrystallized grains will be completely released during recrystallization; therefore, the KAM value is inversely proportional to the recrystallized fraction. It is seen that, in the CI sample, about 60% of the deformed grains are replaced by new strain-free grains, whereas the recrystallized fraction increases to 75% for the CA sample. Moreover, the maximum KAM values for CI and CA samples are 4.60 and 2.81, respectively, suggesting that the dislocation density is remarkably reduced in the CA sample.

The main difference between the two modes and conventional heating methods is in the heating speed. The heating speeds achieved in the two modes were much larger than the conventional heating methods, which are heated exclusively through thermal radiation from the heating elements of the furnace. When pulsed current pass through the sample or graphite die, the sample would be heated up with speeds as high as 6 × 10^5^ K·s^−1^. In this case, a large transient thermal compressive stress would be formed in the sample since the thermal expansion lags behind the temperature rise [11]. The maximum thermal compressive stress can be calculated as follows [14]:

(1) $$\sigma_{max}=E\alpha \cdot \Delta T$$

where *E* = 44.8 GPa is the Young’s modulus, *α* = 2.2 × 10^−5^μm·m^−1^·K^−1^ is the thermal expansion coefficient [15], and Δ*T* is the instant temperature increase caused by the pulsed current and can be calculated by the following equation [12]:

(2) $$\Delta T=\frac{j^{2}\rho t_{c}}{C_{p}d}$$

where *j* = 1500 A is the electric current density, *ρ* = 92 × 10^−9^ Ω·m is the resistivity, *t*_c_ = 39.6 ms is the time of the pulsed current, *d* = 1.78 g·cm^−3^ is the density of material, and *C_p_* = 1.05 kJ·kg^−1^·K^−1^ is thermal capacity. The thermal stress was calculated as 432 MPa for the samples, which was much larger than the yield strength of AZ31 (220 MPa), and thus could promote recrystallization at a low temperature within short time.

In addition, the applied pulsed current could provide an additional driving force for atomic diffusion due to electromigration [16,17]. The potential energy, *Q*_e_, is given by [18]:

(3) $$Q_{e}=A\left|Z^{*}\right|e\rho^{\prime }j$$

where *A* is a constant, Z* is the effective valence, *e* is the charge of an electron, *ρ*′ is the resistivity, and *j* is the current density. In this case, the effective diffusion coefficient, *D*_eff_, can be expressed as [18,19]:

(4) $$D_{eff}=D_{0}exp\left(-\frac{Q-Q_{0}}{kT}\right)=D_{0}exp\left(-\frac{Q-Q_{e}}{RT}N_{A}\right)\;=D_{0}exp\left(-\frac{Q’}{RT}+\frac{A\left|Z^{*}\right|e\rho^{\prime }j}{RT}\right)$$

where *R* is the gas constant, *N*_A_ is the Avogadro constant, *Q*′ is the activation energy for a mole of atoms, and *A*_1_ is a constant. Thus, the application of a pulsed current could reduce the activation energy of diffusion and thus enhance the mass transport [20].

It is reported that a moving electron can provide an additional force (electron wind force) on dislocation with an electric current passing through, which increases the dislocation mobility [21] as well as accelerates dislocation annihilation [22].

By comparing the EBSD results of the CI and CA samples, it may conclude that the advantages of pulsed current relative to traditional heating methods should be a result of the high heating rate achievable through the Joule effect. The high Joule heating effect could generate high transient stress in the sample due to the nonsynchronous changes in temperature and thermal expansion, which governs the recrystallization behavior of deformed AZ31. Although the athermal effect of the pulsed current could enhance the defects mobility, it plays a more important role in accelerating the coarsening of the recrystallization grains.

## 4. Conclusions

An experimental investigation of the intrinsic effect of pulsed currents on the recrystallization of deformed AZ31 alloy was carried out using two specially designed ensembles. The two ensemble configurations could force the pulsed current flow only through either the graphite die or the sample, so that the sample was heated either by Joule heating or by Joule heating and athermal effects, respectively. It was found that the athermal effect of pulsed currents could enhance the dislocation mobility and thus accelerate the coarsening of the recrystallization grains. However, high heating speeds, which can generate high transient stress in the sample due to the nonsynchronous changes in temperature and thermal expansion, play a vital role in governing the recrystallization behavior of deformed AZ31B alloy.

## Data Availability

The data presented in this study are available on request from the corresponding author. The data are not publicly available due to restrictions eg privacy.
